# Optimizing the Intensive Care Treatment of Severe and Complicated *Plasmodium falciparum* Malaria in Nonimmune Patients

**DOI:** 10.1155/2020/1628270

**Published:** 2020-11-21

**Authors:** V. B. Chentsov, A. K. Tokmalaev, G. M. Kozhevnikova, A. M. Baranova, E. T. Vdovina, K. C. Emerole

**Affiliations:** ^1^Infectious Diseases Clinical Hospital No. 2, Moscow, Russia; ^2^Peoples' Friendship University of Russia (RUDN University), Moscow, Russia; ^3^Martsinovsky Institute of Medical Parasitology, Tropical & Vector-Borne Diseases, Sechenov First Moscow State Medical University, Moscow, Russia

## Abstract

This study analyses the intensive care treatment of 48 patients admitted to the Intensive Care Unit (ICU) at the Infectious Diseases Clinical Hospital No. 2, Moscow, Russia, between 2007 and 2019, with a severe and complicated form of *P. falciparum* malaria (B50.8 ICD 10). *Objective*. The aim of this study was to improve the intensive care treatment for severe and complicated *P. falciparum* malaria. The treatment strategy implemented was aimed at preventing ischaemia-reperfusion injury to organs, as well as haemorrhagic complications. The ICU Case Management Protocol set up indications for transferring patients to the ICU which provide preventive (prior to the development of renal failure) application of extracorporeal hemocorrection methods (continuous venous-venous hemodiafiltration and plasmapheresis in a plasma exchange mode) and mechanical ventilation under a medically induced coma, given impaired consciousness as the initial symptom of patients. *Results*. Successful treatment outcome in a majority of the patients (93.8%), shorter ICU length of stay (6.67 ± 1.9 days as compared to 94 ± 1.6 before introduction of the protocol), a median parasite clearance time of 37.50 hours (95% CI 36.21–38.18), and a reduced mortality rate from 29.1% to 6.25% support the efficacy of the ICU protocol in managing severe and complicated *P. falciparum* malaria.

## 1. Background

Imported cases of *P. falciparum* malaria from high endemic countries in Africa, Southeast Asia, and in the recent years from South America are recorded annually in the Russian Federation [1].

Review of data on susceptibility of mosquitoes in the USSR to imported strains of malaria parasites revealed that local *Anopheles* mosquitoes are nonsusceptible to *P. falciparum* [2]. This fact confirms the absence of autochthonous *P. falciparum* transmission by local mosquitoes for over 40 years. There were 1086 confirmed cases of *P. falciparum* malaria in the Russian Federation between 2000 and 2019, of which 44 patients died (4%). Moscow city and the Moscow region had the highest incidence of imported cases with 453 reported cases and 13 deaths (2.9%) between 2009 and 2019. Factors found to be associated with severe and complicated *P. falciparum* malaria were absence of chemoprophylaxis by travellers to endemic regions, lack of information on travel history, healthcare delay, and management and diagnostic errors. In multiple cases, medical practitioners implement conventional antimalarial medication without considering the risk of a drug-resistant *P. falciparum* in endemic regions where travellers may have visited [3], which further delays treatment. It should also be noted that some effective first-line antimalarial drugs recommended by the World Health Organization (WHO) are not licensed in the Russian Federation; therefore, doctors are not able to prescribe these effective drugs in needed circumstances. Most of the patients with imported *P. falciparum* malaria are nonimmune Russian citizens (travellers) which explains the rapid development of severe and complicated forms of the disease. The aim of this study was to improve the intensive care treatment for patients with severe and complicated *Р. falciparum* malaria so as to prevent fatal outcomes.

## 2. Materials and Methods

We analyzed the management of 48 patients with severe and complicated *P. falciparum* malaria admitted to the ICU at the Infectious Diseases Clinical Hospital No. 2, Moscow city, in the period from 2007 to 2019. These patients had been managed with a protocol for severe and complicated *P. falciparum* malaria which was developed at the ICU. The ICU Management Protocol for severe and complicated *P. falciparum* malaria consists of modern methods of extracorporeal hemocorrection (continuous venous-venous hemodiafiltration and plasmapheresis in a plasma exchange mode) and mechanical ventilation under a medically induced coma. A control group was studied to determine the efficacy of the ICU Protocol. This group consisted of 24 patients with severe and complicated *P. falciparum* malaria who were managed without the ICU Protocol because they were admitted before the Protocol was developed (period from 2003 to 2007). All patients had received conventional antimalarial medications. The characteristics of the two groups are presented in Table 1.

**Figure 1:**
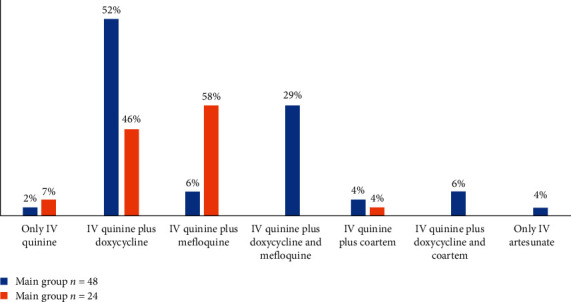
Antimalaria drugs used in patients with severe and complicated *P. falciparum* malaria.

All patients were evaluated under the WHO criteria for severe malaria [4]. It was observed that the parasitaemia level does not always correlate with the disease severity, which can be explained by parasite sequestration in deep capillaries; therefore, peripheral parasitaemia may inaccurately reflect the true burden of the disease. The time from illness onset was vital: day 5 from the illness onset was critical and was regarded as “late admission.” This observation has been cited in other sources [5]. Most patients in the main group presented with cerebral manifestations (Glasgow scale criteria were used to determine the degree of impaired consciousness and coma). In the main group, 40 patients (83.2%)) had signs of multiple organ failure syndrome, 39 (18.7%) had bleeding/disseminated intravascular coagulation, 4 (8%) had hyperbilirubinemia, and 2 (1%) had acute respiratory distress syndrome. Intravenous (IV) quinine was mainly the antimalaria drug indicated (Figure 1). The doses and duration were in accordance with the WHO 2010 guidelines (reviewed and updated in 2016) [6, 7]. IV quinine was ceased after the disappearance of the asexual blood parasites and when the patient could switch to oral treatment after clinical improvement. Such approach reduced the risk of adverse events. IV quinine was combined (and supplemented) with doxycycline followed by oral mefloquine or coartem. Although artesunate remains unlicensed in the Russian Federation, it was used in only one patient in the main group after a collegial decision by the intensivists and infectious disease clinicians due to an established moderate quinine intolerance. In this case, the antimalaria drug (artesunate) was the patient's own medicine used within the ICU.

The treatment strategy was to prevent ischaemia-reperfusion injury to organs, as well as to prevent haemorrhagic complications. After admission to the ICU, central venous catheterization was performed for parenteral administration of drugs. Ischemia and hypoxia have been implicated in cerebral malaria (CM); therefore, oxygen delivery was executed, and hydration was maintained. The ICU Protocol included the utilization of extracorporeal hemocorrection and mechanical lung ventilation. An indication for these procedures was late admission (>5 days from the illness onset), regardless of the level of parasitaemia. Patients showing signs of cerebral malaria (altered mental status) were a strong indicator for mechanical ventilation. This procedure was executed under anaesthesia-inducing drugs (propofol + midazolam). Mechanical ventilation was performed in 20 (41.6%) patients in the main group who presented with altered mental status and impaired consciousness. Extracorporeal hemocorrection involved the use of continuous veno-venous hemodiafiltration and plasmapheresis in a plasma exchange mode. A second large bore two-way IV set was inserted into the femoral vein for extracorporeal hemocorrection procedures. Continuous veno-venous hemofiltration was performed in 37 (77.1%) patients of the main group, using the PrismaFlex system or multiFiltrate devices (Fresenius Medical Care).

Plasmapheresis was introduced in the Protocol in 2014 and was performed in 14 patients of the main group (29.2%). One of the main indications for plasmapheresis was high parasitaemia and metabolic disorders. Intravenous (IV) infusion of 20% mannitol (0.2 g/kg body weight) plus 20 mg furosemide was indicated for cerebral oedema. During the process of fluid infusion, plasma and urine osmolarity, plasma acid-base and electrolyte equilibrium, plasma albumin, and central venous pressure were monitored. The volume of parenteral fluid infused was determined by the volume of enteral nutrition and renal and extrarenal loss. Blood glucose was monitored every 4 hours. Hypotonic solutions and dextrans were not utilized. We managed patients with antioxidants and antihypoxants and implemented prophylactic infusion of fresh frozen plasma to prevent haemorrhagic complications. Clinical and laboratory monitoring of the patients' condition included a complex of clinical biochemistry blood tests, urine test, and other biological specimen and the use of instrumental devices based on indications, echocardiography, chest radiograph, ultrasound of the abdominal, and retroperitoneal and pleural cavities.

## 3. Results and Discussion

**Figure 2:**
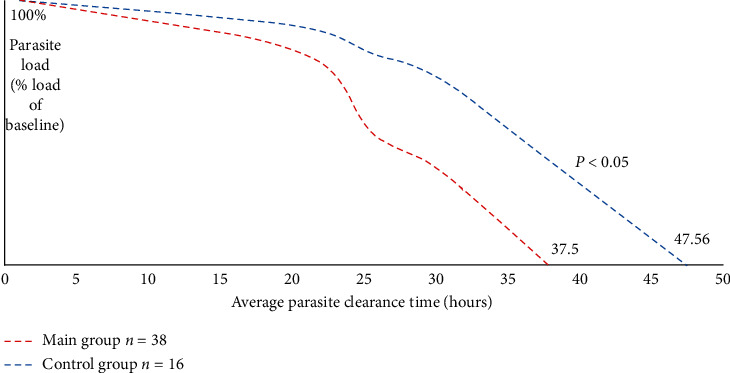
Parasite clearance curve according to mixed model analysis of 54 ICU patients with severe *P. falciparum* malaria.

Imported cases of severe *P. falciparum* malaria in nonendemic regions are common, and there is a high risk of complication in nonimmune individuals from these regions. *P. falciparum* malaria parasites are increasingly resistant to antimalarial drugs; therefore, besides provision of antimalarial drugs, medical experts in nonendemic countries implement strategies to improve intensive care and utilize adjuvant therapies to reduce mortality [5, 8–13]. Previous studies have questioned the effectiveness of additional intensive care methods for treatment of severe malaria and other infectious diseases cases, and we drew attention to the divergent views on this issue [8, 12, 13]. From our standpoint, this may be because methods and their combinations implemented were insufficiently compared. The ICU Management Protocol for severe and complicated *P. falciparum* malaria was updated in 2014 after the introduction of plasmapheresis for intensive care management, given the issue that life-threatening state and complications could occur even after elimination of malaria parasites. Nevertheless, it should be noted that any therapeutic approach based on the use of antimalarial drugs and interventions to correct impaired functions of organs and systems does not always ensure a successful treatment of severe and complicated *P. falciparum* malaria. We evaluated the efficacy of extracorporeal hemocorrection methods in severe malaria by determining the asexual parasite clearance. The parasite clearance estimator (PCE) developed by the Worldwide Antimalarial Resistance Network (WWARN) seemed to be an accurate and reliable method [14]. Out of the overall 72 ICU patients from the two groups, data from 54 ICU patients were suitable for estimation of parasite clearance: the main group had 38 (70%) patients while the control group had 16 (30%) patients. The median parasite clearance time was 37.50 (95% CI 36.21–38.18) hours and 47.56 (95% CI 46.26–48.70) hours for patients in the main group and control group, respectively, *p* < 0.05 (Figure 2). This reveals that extracorporeal hemocorrection contributes significantly to parasite clearance in ICU patients treated with conventional antimalarial drugs, further proving the efficacy of our approach although there was no significant difference in the slope half-life of the median parasite clearance rate: the main group had a slope half-life of 3.12 (95% Cl 1.91–4.88) hours and 4.00 (95% Cl 2.14–5.20) hours for patients in the control group, *p* = 0.671. The duration of illness for all patients ranged from 8 to 41 days (20 ± 2.32 days on average).

*P. falciparum* malaria infection influences blood coagulation by various interacting pathobiological mechanisms, the most important being the overwhelming response of the host to sepsis resulting in a cytokine storm. In addition, the parasite infects the red cells leading to changes in the red cell phospholipid composition which supports blood coagulation. Red cells infected with *P. falciparum* also adhere to the deeper tissue capillary endothelium leading to profound damage to endothelial cells leading to further activation. This results in widespread consumption of platelets and activation of blood coagulation which, at times, culminates in a clinically and pathologically detectable disseminated intravascular coagulation (DIC). Manifestations of blood coagulation disorders are recorded in about 5% of patients with severe *P. falciparum* malaria. We recorded 3 cases of blood coagulation disorders in the main group, 2 of which had died. The transfusion of whole blood and other blood components, red cells, and platelet concentrate remains the most effective treatment for blood coagulation disorders. Thirty-seven (77.1%) patients received plasma and other blood components, 9 patients (18.8%) received transfusion of whole blood and/or RBC, and 6 (12.5%) received platelet concentrate. The protocol was aimed at restoring impaired homeostasis, and the theoretical insight of the procedures involves the removal of a wide variety of biologically active substances (parasite antigens, excess interleukins, tumour necrosis factor, and other cytokines). Numerous studies have implicated a dysregulated immune response and cytokine balance to severe *P. falciparum* malaria [15–17], and this vital factor was demonstrated in one of our previous research [18]. Hemodiafiltration procedures can remove molecules with a molecular weight up to 50 kDa. Continuous venous-venous hemodiafiltration supplemented with plasmapheresis aids the removal of plasma with fragments of parasites; the removal of toxic substances regardless of the presence of free radicals; and the removal of the excess amount of “free haemoglobin” that accumulates during parasite haemolysis. These toxic substances could induce kidney damage when excreted; therefore, the procedures, to a large extent, prevent kidney damage. The removal of huge “toxic stress” restores the immune system's ability to mobilize a more effective response to invading infectious agents which can be vital for the patient in severe malaria cases. Clinical performance and laboratory monitoring revealed that an average duration of 4.68 ± 1.12 days of continuous venous-venous hemodiafiltration therapy and 2-3 sessions of plasmapheresis were sufficient to engender positive outcomes.

Eventually, 45 out of 48 patients recovered fully (93.75%) in the main group. Three deaths were recorded in the main group (6.25%), 7 deaths recorded in the control group (29.1%) with 14 fully recovery, the main group had a shorter ICU length of stay of 6.67 ± 1.9 days compared to (9.4 ± 1.6 days) the control group. These outcomes demonstrate the efficacy of the approach implemented.

We present a case of severe *P. falciparum* malaria managed under the ICU Management Protocol for severe and complicated *P. falciparum* malaria: A 49-year-old man presented to our hospital at the ICU on March 30, 2020, with persistent fever and fatigue for 10 days. A day before, a local hospital had identified *P. falciparum* malaria parasites in his blood smear. The patient noted that a high fever (38.5°C) began 4 days before his return from Zimbabwe on the 21^st^ of March. He did not take antimalarial prophylaxis in Africa. His condition was considered severe with a score of 13 on the Glasgow Coma Scale (GCS). Vital signs were as follows: a temperature of 38.9°C, heart rate of 120 beats/min, blood pressure of 100/70 mmHg, and respiration rate of 28 breaths/min. The patient appeared acutely ill with generalized weakness and drowsiness. His abdomen was soft and distended. The neurological examination was normal although he faintly responded to verbal commands. On examination of blood smears, parasite load for *P. falciparum* was 1846720 mcL at admission and was diagnosed with severe *P. falciparum* malaria: cerebral forms and multiorgan failure. The severity of the patient's condition is due his nonhistory of malaria (nonimmune), late admission, high parasitaemia, and clinical presentation at admission. Laboratory results were as follows: complete blood count revealed a white blood cell (WBC) count of 9.2 per microliters of blood, haemoglobin of 14.2 g/dL, and a platelet count of 11 per microliter of blood. Urinalysis showed protein (+) in the urine. Hepatorenal impairment was present with an aspartate aminotransferase level of 113 IU/L, alanine aminotransferase level of 65 IU/L, total bilirubin (T-bili) of 94 micromol/L, direct bilirubin (D-bilirubin) of 41 micromol/L, blood urea nitrogen (BUN) of 18.8 micromol/L, and creatinine of 156 micromol/L. An ultrasound of the abdomen demonstrated hepatosplenomegaly, haemangiomas of the liver, and deformation of the gallbladder. After admission to the ICU, central venous catheterization was performed for parenteral administration of medications. He was intubated and ventilated due to cerebral manifestations. From the first hours of admission at the ICU, antimalarial therapy (quinine 1 g/daily) and 0.2 g of doxycycline daily were initiated. He was monitored every day for thrombocytopenia. His central venous pressure, acid-base, and hydration status were also monitored. Infusion of fresh frozen plasma and platelet transfusion were initiated. Given the patient's severe status, we initiated our protocol for adjuvant treatment of severe malaria. Our patient required 8 sessions of continuous veno-venous hemodiafiltration (AN69 polysulfone Baxter membrane) from 03/31/2020 to 04/07/20 and 4 sessions of plasmapheresis in a plasma exchange mode (Plasma filter membrane PFM-500) from 03/31/2020 to 04/03/20. After 3 days of quinine intervention, anuria (nonpassage of urine) and symptoms of acute kidney injury increased. Laboratory examination revealed urea nitrogen (BUN) of 53.7 micromol/L, creatinine of 703 micromol/L, serum potassium elevation of 6.4 mmol/L, and haemoglobin of 14.2 g/dL. Echocardiography showed pericardial effusion. An ultrasound of the abdomen demonstrated oedema of the surrounding gallbladder tissue. We ceased quinine treatment due to possible renal failure after erythrocyte haemolysis and switched to artesunate. After 5 days of treatment at the ICU, his peripheral blood smears did not contain any ring-form trophozoites anymore. In the absence of blood parasites, it was decided to stop artesunate treatment and switch to 80 mg coartem. Our patient remained on ventilator therapy for 8 days and developed ventilator-associated pneumonia (VAP) for which he was treated with meropenem for 10 days. By the 10^th^ day of illness, the level of urea and creatinine stabilized, and, given his stable state, he was transferred from the ICU to the infectious general ward for further treatment and observation. By the 19^th^ day of stay at the hospital, the patient's clinical outcome improved and his appetite returned to normal. There were no complications from malarial infection. Haemoglobin, bilirubin, and liver enzyme levels returned to normal. He was discharged after 21 days.

## 4. Conclusions

A combination of conventional antimalarial drugs and adjuvant intensive therapeutic interventions in severe malaria cases could reduce mortality cases. It is on this note that the authors developed and implemented a protocol for managing severe cases of *P. falciparum* malaria which has been in use for over 10 years together with the WHO guidelines (2010 and 2015) for treatment and management of severe malaria cases. This protocol provides preventive (prior to the development of renal failure) application of extracorporeal hemocorrection methods (based on continuous venous-venous hemodiafiltration and plasmapheresis in a plasma exchange mode) and mechanical ventilation under a medically induced coma, given impaired consciousness as the initial symptom of most patients admitted to the ICU. After implementing the protocol, 93.75% of patients with severe and complicated *P. falciparum* malaria recovered and had an average ICU stay of 6.67 ± 1.9 days. The mortality rate of severe *P. falciparum* malaria was reduced to 6.25% from 29.1% before the protocol was introduced which largely demonstrates its efficacy. The procedures and interventions involved were introduced with the key intention of saving the lives of patients, though quite expensive. Therefore, it is not considered in financing programs and measures to combat malaria. The outlined ICU Protocol would gain the attention of medical practitioners, in terms of organisation (logistics). Cases of severe malaria can be avoided by adhering to general recommendations and measures which include observing chemoprophylaxis when traveling to endemic regions, prompt diagnosis, and adequate treatment of patients. The authors note the need for systematic training and education of medical practitioners including acquiring the knowledge of drug-resistant parasites. Health recommendations and general travel advice for international travellers to endemic regions should be pursued. Another vital strategy which the authors support is to increase accessibility and enforce license of effective first-line antimalarial drugs.

## Figures and Tables

**Table 1:** General characteristics of patients with imported severe and complicated *P. falciparum* malaria.

	Main group	Control group	*P* value
Number of patients (*n*). Men/women (*n*). Median age, years (range)	48. 38/10. 41.3 ± 3.97 (16–69)	24. 22/2. 43.2 ± 2.37 (21–58)	0.033. 0.034
Nationality			0.013
Russian citizens (*n*, %). Immigrants travelling or residing in Russia	44 (91.7%). 4 (8.3%)	23 (96.6%). 1 (3.4%)	
Disease-endemic area visited: Africa (*n*, %), India (*n*, %), and South America	44 (91.7%). 1 (2%). 3 (6.3%)	23 (96.6%). 1 (3.4%)	0.059
Time from illness onset to hospital admission, days (average)	(7.5 ± 1.17)	(6.0 ± 1.5)	0.047
Duration of hospital stay, days (range)	3–21	4–19	0.072
Level of parasitaemia at admission, (*μ*l) blood (average)	2040–1087800 (375529 ± 408)	5780–1002100 (299247 ± 320)	0.050
Underlying disease (*n*, %)	11 (22.9%)	3 (12.5%)	0.089

*Note*. All *P* values for categorial variables were calculated using chi-square tests. All *P* values for continuous variables were calculated using Wilcoxon rank-sum tests.

## Data Availability

Data can be made available upon request.
